# Atomic and vibrational origins of mechanical toughness in bioactive cement during setting

**DOI:** 10.1038/ncomms9631

**Published:** 2015-11-09

**Authors:** Kun V. Tian, Bin Yang, Yuanzheng Yue, Daniel T. Bowron, Jerry Mayers, Robert S. Donnan, Csaba Dobó-Nagy, John W. Nicholson, De-Cai Fang, A. Lindsay Greer, Gregory A. Chass, G. Neville Greaves

**Affiliations:** 1 Department of Oral Diagnostics, Faculty of Dentistry, Semmelweis University, Budapest 1088, Hungary; 2 Department of Electronic and Electrical Engineering, University of Chester, Thornton Science Park, Chester CH2 4NU, UK; 3 School of Electronic Engineering and Computer Science, Queen Mary University of London, London E1 4NS, UK; 4 Laboratory of Extreme Glassy State, State Key Laboratory of Silicate Materials for Architectures, Wuhan University of Technology, Wuhan 430070, China; 5 Department of Chemistry and Bioscience, Aalborg University, DK-9220 Aalborg, Denmark; 6 ISIS Facility, STFC Rutherford Appleton Laboratory, Chilton, Didcot, Oxon OX11 0QX, UK; 7 School of Sport, Health and Applied Science, St Mary's University, London TW1 4SX, UK; 8 College of Chemistry, Beijing Normal University, Beijing 100875, China; 9 Department of Materials Science and Metallurgy, University of Cambridge, 27 Charles Babbage Road, Cambridge CB3 0FS, UK; 10 School of Biological and Chemical Sciences, Queen Mary University of London, London E1 4NS, UK; 11 Department of Physics, Institute of Mathematics, Physics and Computer Science, Aberystwyth University, Aberystwyth SY23 3BZ, UK

## Abstract

Bioactive glass ionomer cements (GICs) have been in widespread use for ∼40 years in dentistry and medicine. However, these composites fall short of the toughness needed for permanent implants. Significant impediment to improvement has been the requisite use of conventional destructive mechanical testing, which is necessarily retrospective. Here we show quantitatively, through the novel use of calorimetry, terahertz (THz) spectroscopy and neutron scattering, how GIC's developing fracture toughness during setting is related to interfacial THz dynamics, changing atomic cohesion and fluctuating interfacial configurations. Contrary to convention, we find setting is non-monotonic, characterized by abrupt features not previously detected, including a glass–polymer coupling point, an early setting point, where decreasing toughness unexpectedly recovers, followed by stress-induced weakening of interfaces. Subsequently, toughness declines asymptotically to long-term fracture test values. We expect the insight afforded by these *in situ* non-destructive techniques will assist in raising understanding of the setting mechanisms and associated dynamics of cementitious materials.

## Abstract (web summary)

Bioactive cements are widely used in dentistry and medicine. Here, using a variety of novel experimental techniques, the authors uncover the atomic and vibrational contributions to the mechanical toughness of bioactive cement during the setting process.

## Graphical abstract

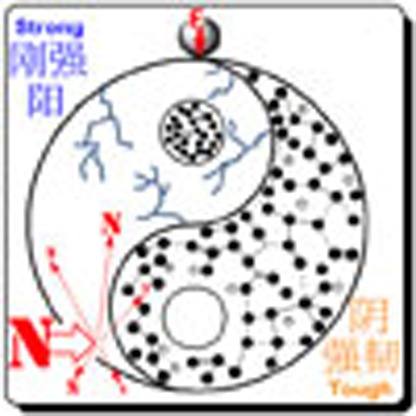

Worldwide demand for durable biomaterials emanates from population ageing and from emergent developing countries. Historically, the implantation of foreign materials into the body has been dentistry led. However, the optimisation of tooth replacements remains incomplete, problems stemming from conflicts between mechanical toughness, biocompatibility, adhesion and appearance. For commonly used mercury–silver amalgams, this is compounded by toxicity and disposal. The United Nations Environment Programme assesses mercury to be ‘a global threat to human and environmental health,' listing amalgams as a source^1^. With 125 million amalgam restorations carried out annually in Europe, the European Commission advocates atraumatic restorative treatment using mercury-free alternatives^2^, highlighting glass ionomer cements (GICs) (Fig. 1a) as an excellent option. Developed over 40 years^3^,^4^,^5^,^6^,^7^,^8^, GICs are the product of a basic fluoro-phospho-alumino-silicate glass powder and an aqueous poly(acrylic) acid (PAA) solution (Fig. 1b; Methods)—also known as glass polyalkenoate cements^9^. Although cost-effective and environmentally friendly^2^, caries-resistant and bioactively mineralizing dentine^10^,^11^, GICs remain too brittle for permanent implants^12^,^13^. With exceptional bonding to the apatite phase of bone, GICs have also been considered for other aspects of surgery^14^,^15^,^16^, but necessarily confined to non-load-bearing applications with moderate durability requirements^17^.

Damage tolerance is assessed through fracture toughness *K*_C_ and yield strength *σ*_Y_, both traditionally measured by destructive methods^18^. Average values for dental materials and their components have been collated to create Fig. 1c. This log *K*_C_ versus log *σ*_Y_ plot follows the Ashby scheme^19^ widely used elsewhere in mechanics to categorize conflicts between strength and toughness in composite materials^20^. Polymers and ceramics lie in the lower half with *K*_C_ values greater than 1 MPa m^1/2^; brittle materials such as glasses have *K*_C_ values less than this. Indeed the yield strength *σ*_Y_ of glassy materials covers many decades^21^ from ∼10 GPa (glass fibres) to ∼1 MPa (pre-damaged glass). Respective *K*_C_ and *σ*_Y_ values of GICs cluster around those of dentine and amalgam, but clearly have less toughness and strength than either.

For composites in general, and GICs in particular, *K*_C_ and *σ*_Y_ develop during setting, starting out as highly deformable and incompressible slurries that subsequently harden to form rigid, inflexible cements. The compressibility *κ* relates directly to the shape of the interatomic potentials of a given system^22^. The narrower and deeper the potential is, the stronger and more rigid the atomic cohesion and vice versa. More rigid materials have a higher shear modulus *G* and lower *κ*, but most importantly tend to have a higher *G·κ*, and to be brittle. In particular, Poisson's ratio *ν* (ref. 22), a function of *G·κ*, sharply differentiates between brittleness and ductility. If toughness is converted to fracture energy *G*_C_ (fracture energy *G*_C_ is related to fracture toughness *K*_C_ by 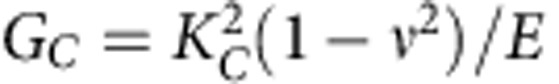, where *E* is Young's modulus and *ν* is Poisson's ratio), as a function of *ν*, this forms a clear sigmoid, with an inflection point (at *ν≈*0.33)^23^ separating ductile (*ν*>∼0.33) from brittle (*ν*<∼0.33) materials (Fig. 1d). Originally discovered for metals^23^, this important empirical relationship also holds for inorganic glasses and polymers, the ingredients of GICs and other non-metals. All these materials have been incorporated into Fig. 1d to create a guide for assessing the extent of brittleness of dental materials. In particular, as setting advances, *ν* must decrease significantly, from *ν*≈0.5, when the cement is a virtually incompressible liquid to *ν*<∼0.33, as it solidifies into a brittle solid. This is illustrated by the dashed arrow for GICs, which finally become too brittle, with *ν≈*0.30 (ref. 24) compared with amalgam with *ν≈*0.34 (refs 22, 25). The ultimate aim is to modify GICs so that they are closer in toughness to amalgam and dentin.

The initial setting mechanism of GICs is an acid–base reaction between the aqueous PAA and the glass component (Fig. 1b; Methods)^3^,^4^. As with alumina-silicate glasses, water corrosion ruptures bridging oxygens (BOs) to form SiOH (ref. 26) and AlOH (ref. 27) groups, initially creating an aqueous gel at the glass surface. For the G338 glass used in GICs, PO_4_^−^ and F^−^ will also be released, along with Na^+^ cations, freeing Al^3+^ and Ca^2+^ cations to crosslink the polymer to form a strong polysalt matrix^4^,^5^. Aluminium chelation by the polymer drives the conversion of Al(IV) tetrahedra to higher-coordinated sites^28^ at the interfaces between glass and the matrix, as well as those between crosslinked polymer chains within the matrix. Higher-coordinated sites include both pyramidal Al(V) as well as octahedral Al(VI) geometries. Similar changes in interfacial configuration in proteins, for example, are manifested by variations of orientation-dependent dynamics in the sub-THz range^29^. These low-frequency modes are known to modulate mechanical^30^, optical^31^ and biophysical properties^32^ of macromolecular systems.

We have therefore turned to non-destructive techniques that record changing atomic structures, associated collective sub-THz dynamics and atomic cohesion during the first 3 days of setting, uncovering highly nonlinear behaviour over various stages, and providing indications of the sources of eventual brittleness and low strength.

## Results

### Heterogeneous glass

**Figure 1:**
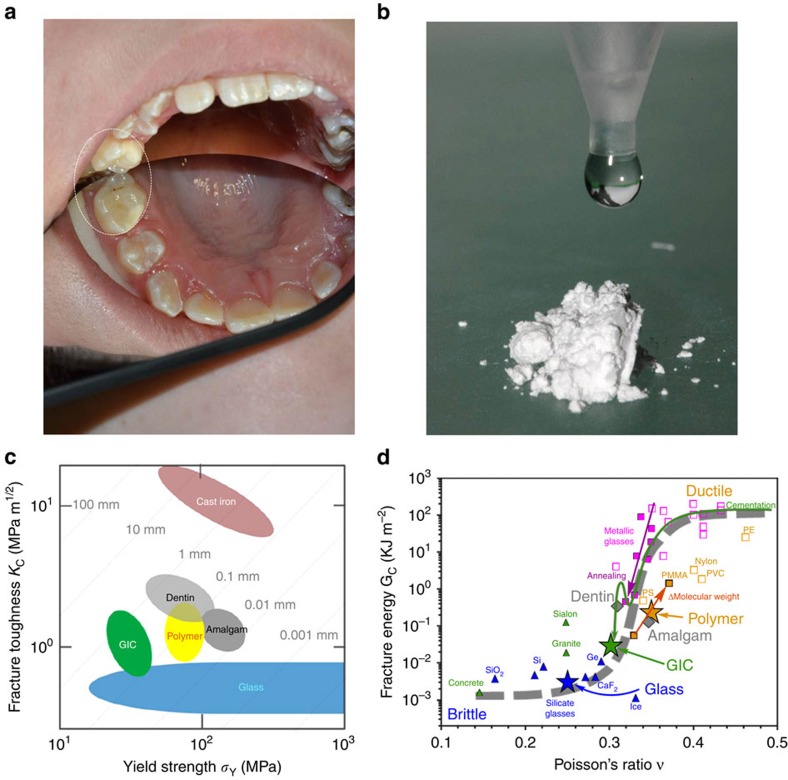
GICs properties and glass nanoscopic structure. (**a**) GIC restorative, including occlusal view. (**b**) G338 fluoro-alumino-silicate glass powder and dangling aqueous polymer (acrylic acid). (**c**) Fracture toughness *K*_C_ versus strength *σ*_Y_ for dental materials compiled as an Ashby plot^19^: dentin, glass and polymers^20^,^21^,^45^, GICs^46^,^47^ and amalgam^60^. (**d**) Fracture energy *G*_c_ versus Poisson's ratio *ν* and the brittle–ductile transition^22^,^23^, expanded for a wide range of materials; *ν* values for dental materials^24^,^25^,^61^,^62^ combined with *G*_C_ values^20^,^21^,^22^,^23^,^45^,^46^,^47^,^60^,^61^; toughness decline during setting indicated by the dashed arrow; trends for annealing and polymerization (solid arrows). **a** and **b** courtesy Faculty of Dentistry, Semmelweis University.

**Figure 2:**
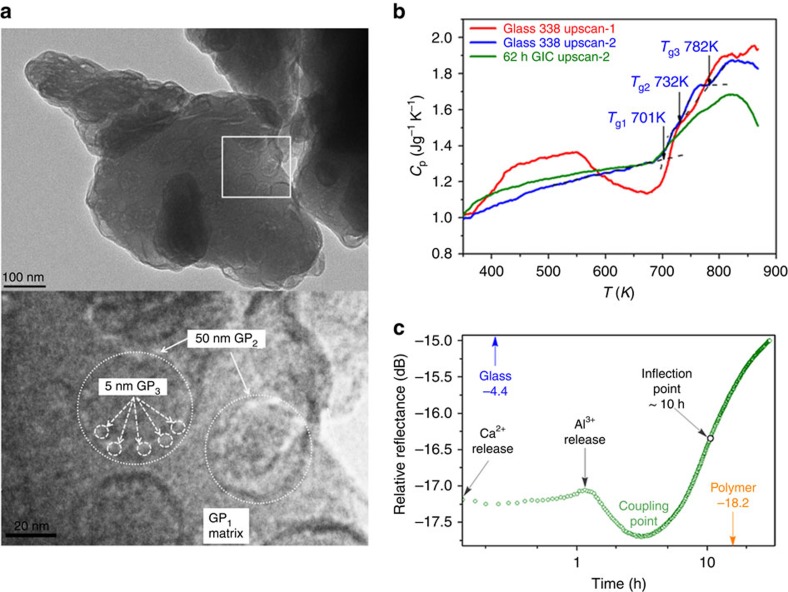
GIC characterization. (**a**) TEM image of individual glass particle (above, scale bar, 100 nm) with three distinguishable glass phases: GP1, GP2 and GP3 seen on an expansion of the white frame area (below, scale bar, 20 nm, see text for details). (**b**) Three DSC upscan curves for the fresh G338 glass (red), the G338 glass subjected to the first up- and downscans (blue), and the GIC sample (green) subjected to 62 h setting and the subsequent up- and downscans, respectively. The red curve exhibits a water-loss endothermic response, followed by an exothermic enthalpy-release response; the blue upscan-2 curve reveals three sharp glass transitions *T*_g1_, *T*_g2_ and *T*_g3_, which we associate with the glass phases GP1, GP2 and GP3, respectively; the green curve reveals how glass transitions are modified by setting. Both up- and downscan rates are 20 K min^−1^. (**c**) Coherent terahertz spectroscopy: changing sub-THz relative reflectance during setting, differentiating gel formation (Ca^+2^ and Al^3+^release) from chelation (Al^+3^ release); minimum (CP) identifies the point where glass and polymer couple dynamically.

To make our extended *in situ* experiments relevant to current dental practice, we have chosen the commercial G338 ionomer glass (Fig. 1b), in regular use since the early 1980s. To ensure practical relevance, we employed standard clinical preparatory mixing to examine setting from the polymer–glass mixture to the hardened cement. The glass powder has been imaged by transmission electron microscopy (TEM) and exhibits significant heterogeneity, with three glass phases (GPs) identifiable on the scale of 5–50 nm (Fig. 2a). Figure 2b and Supplementary Fig. 1 show the isobaric heat capacity *C*_p_ of both G338 and GIC samples as a function of temperature determined by differential scanning calorimetry (DSC). The *C*_p_ of fresh G338 sample (red curve) exhibits the effects of water loss, after which (blue curve) three sharp glass transitions can be deciphered. Following 62 h of setting, the *C*_p_ of the GIC (green curve) demonstrates the evolution of the glass-transition regions of the remaining glass.

Coherent terahertz spectroscopy (CTS)^33^ has been employed to track changes in inter-particle binding at the interfaces through variations in collective low-frequency atomic dynamics during setting. Non-monotonic behaviour is clearly evident, revealing several large swings in the magnitude of THz dynamics (Fig. 2c).

**Figure 3:**
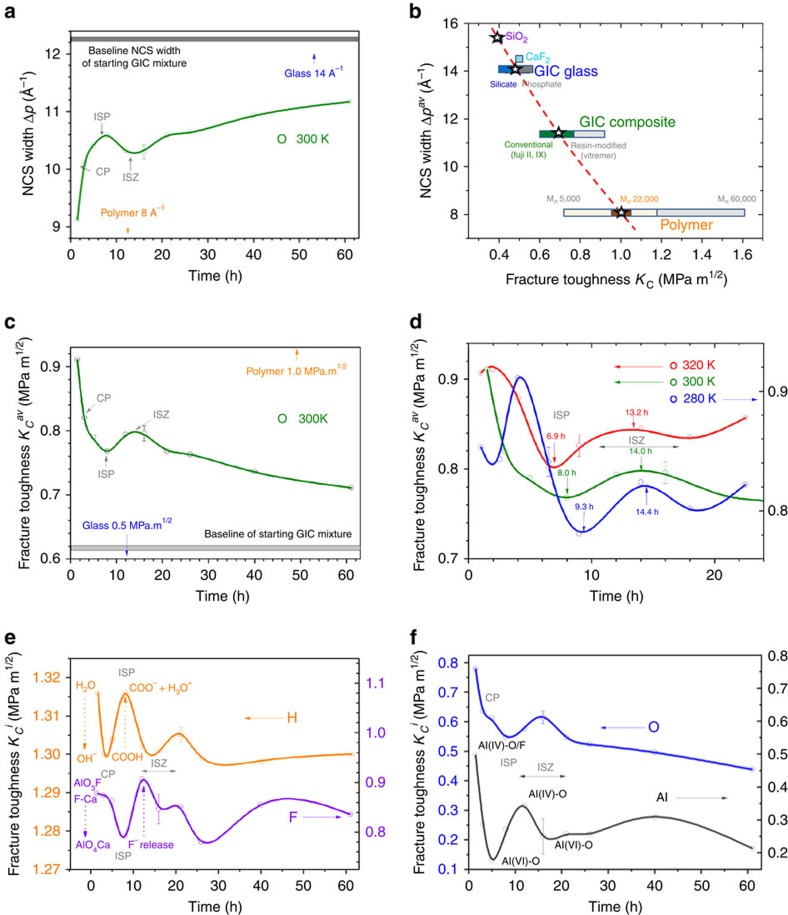
Non-monotonic advancement in atom cohesion and fracture toughness during GIC solidification. (**a**) Overall GIC NCS peak width Δ*p*^av^ variations with setting time, measuring different stages in atomic cohesion during setting: CP, ISP and ISZ (see text for details). (**b**) Inverse relationship between atomic cohesion Δ*p*^av^ and fracture toughness *K*_C_^av^ of GIC, polymer and glass (asterisks) and associated materials (see text for details). (**c**) Non-monotonic fall in overall *K*_C_^av^ with setting time at 300 K obtained from overall Δ*p*^av^ **a** using **b**, identifying CP and reaction points ISP and ISZ. (**d**) *K*_C_^av^(280 K), *K*_C_^av^(300 K) and *K*_C_^av^(320 K), showing shifts in ISP and ISZ with setting temperature. (**e**) Fluctuations in *K*_C_^i^ for H and F, showing evidence for hydration and fluorination. (**f**) *K*_C_^i^ for O and Al through the various setting stages. Elemental fracture toughness *K*_C_^i^ were derived from Δ*p*_i_ values (Supplementary Fig. 3a), with *K*_C_^av^ (280, 300 and 320 K) obtained from Δ*p*^av^ (280, 300 and 320 K) values (Supplementary Fig. 3b), in each case using **b**.

For observing mechanical toughness *K*_C_ atomically, we have turned to *in situ* neutron Compton scattering (NCS)^34^, where neutron momentum recoil Δ*p* measures the atomic cohesion. The development of Δ*p* during setting at 300 K is clearly oscillatory (Fig. 3a; Supplementary Fig. 2). Using a new empirical relationship between the momentum recoil Δ*p* values and published fracture toughness values (Fig. 3b), changes in atomic *K*_C_ have been analysed both for the total system *K*_C_^av^ (Fig. 3c,d) and for separate elements *K*_C_^H,F,O,Al^ (Fig. 3e,f).

**Figure 4:**
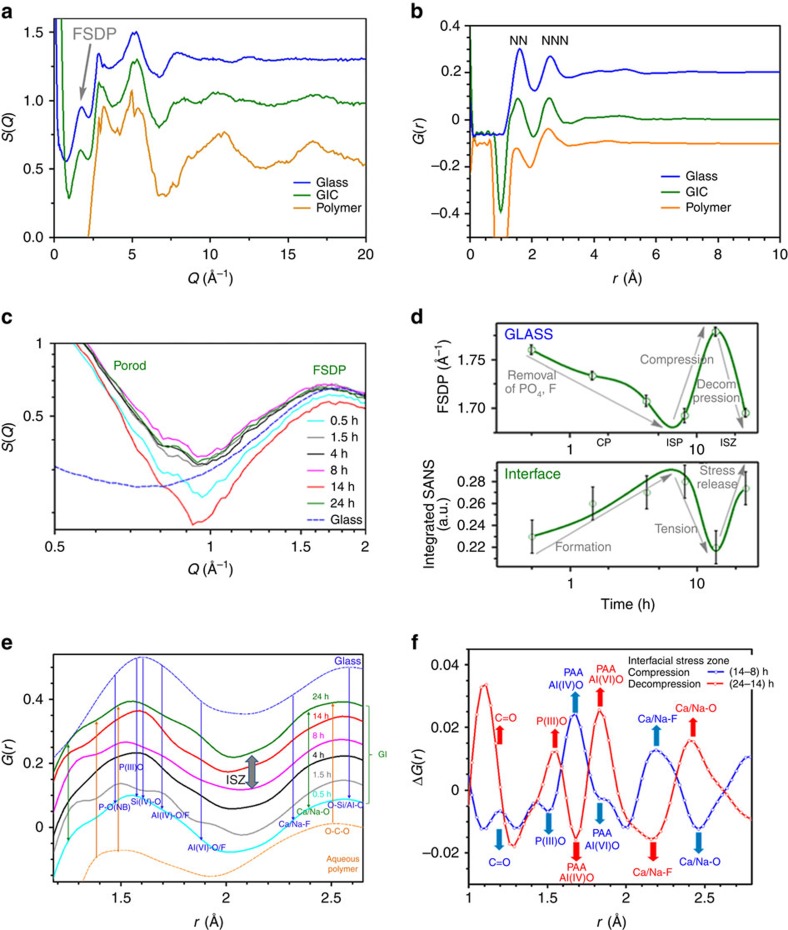
Neutron scattering measurements. Time-averaged (**a**) *S*(*Q*) and (**b**) *G*(*r*), including FSDP and expected locations for nearest- and next-nearest-neighbour pair correlations NN and NNN, respectively. (**c**) *In situ* time-resolved *S*(*Q*) over 24 h revealing changes in small-angle Porod scattering (SANS) at interfaces, and in the position of FSDP for the glass. (**d**) Fluctuations with setting in the FSDP position and the integrated SANS from **c** (Methods). Abrupt changes around 15 h coincide with the Δ*p*^av^ minimum and *K*_C_^av^ maximum (Fig. 4a,c). (**e**) *In situ* time-resolved *G*(*r*) with NN distances for GIC related to glass (upper) and aqueous polymer (lower). (**f**) Trends in Δ*G*(*r*) obtained by differencing *G*(*r*) across the ISZ (from **d**)—splines for guiding the eye. **a**,**b** and **e** data are all offset vertically for clarity of presentation.

*In situ* neutron scattering measurements also reveal complementary variations in the structure factor *S*(*Q*)^35^ associated with nanoscopic structure changes taking place during setting (Fig. 4a,c), and in the real-space transform *G*(*r*) (Fig. 4b,d,e). This extensive set of *in situ* experiments has been used to quantify mechanical, structural and dynamical parameters during the setting of GICs, previously unobtainable atomistically^7^.

The G338 glass is chemically complex, containing ingredients for cementation (calcium, phosphate and alumino-silicate), mineralization (phosphate and fluoride), dental caries resistance (fluoride) and opal appearance (Ca+F-rich particles). The network-forming ions are Si^4+^, P^5+^, with the majority being Al^3+^. Al^3+^ can be charge-compensated for tetrahedral configuration by P^5+^, and also by Na^+^ and Ca^2+^, which, as network-modifiers, can promote the formation of non-BOs within the BO alumina-silicate network^4^,^36^. Considerable fluorine content further depolymerizes the glass melt, leading to low liquidus temperatures^28^. In the glass, Al^3+^ coordinates both to BOs and F^−^ (ref. 36), while F^−^ complexes with Ca^2+^ and Na^+^ as well as with Al^3+^. SiO_4_ and PO_4_ tetrahedra principally link via BOs to Al polyhedra, and, while these are mainly tetrahedral, Al(V) and Al(VI) configurations also occur, particularly at the developing interfaces, with the proportions changing during cement setting^28^,^36^.

The as-received G338 glass powder (Methods) comprises micron-sized particles (Fig. 2a) exhibiting extensive amorphous phase separation, as others have reported^17^,^37^. Our high-resolution TEM image includes a continuous matrix (GP_1_), in which are embedded 30–50-nm spherical rosette domains (GP_2_), decorated by 5–10-nm droplets (GP_3_). GP_2_ and GP_3_ are highlighted by large and small dashed circles, respectively, and are generally seen throughout this image and those of other particles. All three GPs are neutron amorphous (Fig. 4a) and the three glassy states can be verified by DSC traces (Fig. 2b). These reveal three glass transitions: *T*_g1_ (701 K), *T*_g2_ (732 K) and *T*_g3_ (782 K). The small size of GPs and overlaying within TEM images prevents measurement of phase compositions to correlate with *T*_g_s. However, as amorphous phase separation is the primary source for bulk crystalline nucleation in glass ceramics^38^, crystallographic studies of dental ceramics and devitrified GIC glasses^37^ identify possible glass-phase compositions. In particular, G338 glass, which is a typical phosphate-containing fluoro-alumino-silicate glass, exhibits amorphous phase separation resembling the morphology in Fig. 2a and at least two glass transitions at similar temperatures to those appearing in Fig. 2b^37^. Further annealing drives crystallization by bulk nucleation to a fluoro-phosphate phase and an alumino-silicate phase, the former emanating from amorphous phase-separated droplets and the latter from the surrounding matrix^37^,^39^. Accordingly, we attribute the surrounding matrix seen in our TEM images to an alumino-silicate GP (GP1 *T*_g1_) and the nanophase droplets to a Ca-F-P-rich GP (GP3 *T*_g3_). From the glass composition (see Materials in the Methods section) the remaining 30–50-nm spherical domains suggest a Ca-F-rich GP (GP2 *T*_g2_). These attributions to the phase-separated components have been confirmed in a recent parallel study^40^.

Thermodynamically, the as-received G338 glass is far from equilibrium, as evidenced by the broad exothermic peak around 720 K that precedes the glass transitions (Fig. 2b red curve). This shows the release of the enthalpy trapped during the very rapid quenching of the G338 melt^41^ and is absent on reheating (Fig. 2b blue curve). Accordingly, we expect each of the three separated amorphous phases GP_1_, GP_2_ and GP_3_ (Fig. 2a) to be structurally and energetically heterogeneous in the as-received glass, as discovered in other hyperquenched glasses^41^. Unstable phases will help drive the hydration process when PAA and glass are mixed.

By heating the GIC cement above room temperature, DSC reveals, first the release of water and any organic impurities and then the decomposition of the polymer, which coincides with the three *T*_g_s in the annealed glass (Supplementary Fig. 1a). The physical consequences of GIC setting are reflected in the altered glass-transition pattern of the residual glass (Fig. 2b green curve) compared with the annealed G388 glass (Fig. 2b blue curve). Most obviously, the boundaries between all three glass transitions in the GIC cement are less distinct, suggesting that all the phases have reacted with PAA. This is most pronounced for GP2 *T*_g2_ and GP3 *T*_g3_, where the jump from glass to liquid is reduced, which will be partly due to their large surface area. Reactions with PAA might promote a disorder–order transition^41^, or even partial mineralization, both of which would lower the glass-transition peaks. By contrast, the onset temperature of GP1 *T*_g1_ is least affected by setting, indicating that the primary alumino-silicate network remains largely intact during setting.

### Cooperative interfacial dynamics

We observe time-dependent changes in the sub-THz range using CTS^33^ in the early stages as GIC cementation advances (Fig. 2c). These occur between the separate CTS values of the glass and polymer. Since bulk values will not vary, the changes that we see must relate directly to the low-frequency dynamics developing at the interfaces between glass and polymer, as well as those between crosslinked polymer chains within the matrix during setting. The vibrational modes in the sub-THz energy are centred around 0.5 THz and mainly involve collective motions of constituent atoms. These will be increasingly added to, during setting, by inter-component librational changes such as twisting, bending and flexing, with interfacial links serving as pivot points. As these encompassing motions modulate macroscopic interfacial and mechanical properties^29^,^30^,^32^, such as plasticity and elasticity^31^, the initial dip in CTS signal coincides with Ca^2+^ release from the glass. This would appear to be due to the cationic effusion. Governed by the Ca^2+^ rattling frequency in the glass (∼12 THz)^42^, this would initially outpace the polymer's ability to deform rapidly enough to bind the excess ions, being limited by the polymer's intrinsic low-frequency dynamics (∼0.5 THz). Once Ca^2+^ is released from the glass, however, subsequent signal recovery over the first 1 h traces the polymer's progressive chelation of Ca^2+^ at the interface, until Al^3+^ emerges from the glass network at ∼1.5 h, initiated by a sharp drop in reflectance continuing to ∼5 h. As the polymer is increasingly localized into a percolating matrix around the glass powder^13^ and, with gel formation on the glass-particle surfaces^4^,^5^, we would expect an associated dampening of the CTS signal, approaching the values of the isolated polymer component (Fig. 2c). This drop, however, is then followed by a sharp increase in THz reflectance, signalling increased activation of interfacial collective modes, and thus of increased coupling between polymer and alumino-silicate glass components. Accordingly, the minimum at 3 h, we propose, defines a coupling point (CP) in the reaction-setting mechanism, after the cement has lost its initial plasticity (Fig. 1d), but before it has started to establish its mechanical strength. More generally, modulation of fluctuations in the sub-THz-regime generally has been linked to elasticity and shear-induced phase transitions^43^ and has also been associated with the stability of zeolite structures^44^, proteins^32^ and in general is typical of the librational dynamics of two-level systems in network structures^42^.

### Atomic cohesion and fracture toughness changes

The momentum peak widths Δ*p*_i_ of individual atom types *i*, measured by NCS (Supplementary Fig. 3), quantify the depths of interatomic potentials, and these relate directly to atomic cohesion^34^. Marked oscillatory changes in Δ*p*_i_ occur during GIC setting (Supplementary Fig. 3a), particularly over the first day. To obtain the average momentum width Δ*p*^av^ representative of all elements in the setting cement, Δ*p*_i_ are combined as Σ_*i*_c_*i*_Δ*p*_*i*_, where *c*_i_ is the element fraction. Note that Δ*p*^av^ is bounded by Δ*p*^polymer^ and Δ*p*^glass^ widths for respective polymer and glass components, measured separately (Fig. 3a). As setting advances, Δ*p*^av^ starts close to the Δ*p*^polymer^ width, and increases over 62 h, levelling off below the width of the starting mixture, which is dominated by glass Δ*p*^glass^. During this time there is an inflection point at ∼5 h, which we have identified from CTS THz spectra as the CP between glass and polymer. This is followed by a clear maximum at ∼8 h, where the atomic cohesion is greatest. We define this as the initial setting point (ISP). This might be the desirable point for cementation to halt. However, there is then a minimum at ∼15 h, where atomic cohesion momentarily drops before recovering. We later identify this, from changes in *S*(*Q*) (Fig. 4d), as an interfacial stress zone (ISZ). Thereafter, the average momentum width Δ*p*^av^ starts to level out.

Since NCS probes atomic cohesion^34^, when Δ*p*^av^ is lower, average atomic cohesion is also lower, interatomic potentials shallower and wider and thus the material is tougher, and vice versa. Accordingly, we expect that Δ*p*^av^ and fracture toughness *K*_C_ might be inversely related for groups of materials. This is demonstrated in Fig. 3b for the GIC system studied, where Δ*p*^av^ values for this GIC composite measured at 24 h are plotted, together with Δ*p*^polymer^, Δ*p*^glass^ measured separately, as well as those of related compounds (see Supplementary Materials). All are plotted directly as a function of values of fracture toughness *K*_C_ reported in the literature^21^,^45^,^46^,^47^. These extend from single values for specific materials, such as SiO_2_ or CaF_2_, to ranges of values of *K*_C_ for different systems such as glasses and polymers, which span different compositions and material treatments. The asterisked values relate to the particular components, compositions and preparation protocol used in this study (Methods). The spread of the *K*_C_ values around these asterisked points for other glasses, GICs and polymer systems extend to smaller or larger values. These are smallest for oxide glasses and largest for phosphate glasses. Fracture toughness of the GIC glass falls midrange defining the value used in Fig. 3b. Compared with glasses, the span of *K*_C_ is much larger for polymers, where *K*_C_ is strongly governed by molecular weight *M*_n_ (ref. 45), which, for the polymer used for the present GIC, lies close to 22,000 (refs 7, 9) and determines the asterisked value in Fig. 3b. The value of *M*_n_ in turn influences the mechanical properties not just of polymers but also of GICs^48^,^49^. In particular, *K*_C_ is greater for resin-modified GICs than for conventional GICs where the current GIC system falls midway, which determines the final asterisked value. Taken together, these well-defined points result in our empirical relationship between Δ*p*^av^ and *K*_C_ being also well-defined. This is not a reciprocal relationship, as the negative slope, dΔ*p*^av^/d*K*_C_, decreases with increasing *K*_C_. Figure 3b provides a practical look-up table to calibrate Δ*p*^av^ widths in Å^–1^ measured with NCS with fracture toughness in MPa m^1/2^, and is used to convert Δ*p*^av^ from Fig. 3a into average fracture toughness values *K*_C_^av^ at 300 K during setting (Fig. 3c).

Both atomic cohesion measured from Δ*p*^av^ using NCS and fracture toughness *K*_C_^av^ are opposite sides of the same coin and reveal the same CP, ISP and ISZ features in monotonic setting observed early on (Fig. 3a,c). Moreover, *K*_C_^av^ obtained from NCS observations offers a way to continuously access mechanical toughness *in situ*, otherwise complicated by the statistical averaging of multiple specimens inherent in retrospective destructive fracture testing^18^. In particular, *K*_C_^av^ drops sharply with setting time (Fig. 3c), in line with the anticipated fall in *ν* (Fig. 1d), followed by the coupling (CP), setting (ISP) and stress (ISZ) points. Thereafter, *K*_C_ levels out over the next 2 days, significantly above the baseline toughness—derived using the mixing ratio of the glass and polymer components' *K*_C_ values (Fig. 3c). We observe similar progression in *K*_C_^av^ at different setting temperatures over the first day (Fig. 3d; Supplementary Fig. 3b). These reveal expected upward shifts in temperature with time for the features CP, ISP and ISZ, as the setting temperature increases.

The elemental momentum widths Δ*p*_*i*_ (Supplementary Fig. 3a) analysed from the single-particle momentum distribution (Supplementary Fig. 2b) can be converted into elemental fracture toughness values *K*_C_^i^ using the same empirical relationship (Fig. 3b). These are shown for H, F, O and Al (Fig. 3e,f) and share the same overall scale as *K*_C_^av^ (Fig. 3c), ranging from H with the highest fracture toughness values (∼1.3 MPa m^1/2^) to Al with the lowest (∼0.3 MPa m^1/2^). Elemental values can be interpreted as imparting different degrees of fracture toughness at the atomistic level, with H and Al, for example, possibly contributing toughness and brittleness, respectively. The reaction points CP, ISP and ISZ are again evident. However, H, F and Al exhibit oscillatory changes in *K*_C_^i^ (and Δ*p*_i_) with setting time (Fig. 3e,f; Supplementary Fig. 3a), which are averaged out in *K*_C_^av^. These may be associated with developments in hydration (*K*_C_^H^), fluorination (*K*_C_^F^) and chelation (*K*_C_^Al^), respectively. This sensitivity to differing chemical bonding is illustrated by hydrogen (Supplementary Fig. 3a). Eventual Δ*p*^H^ for GICs reported here is 4.81 Å^−1^, similar to water (4.84 Å^−1^), but greater than ZrH_2_ (4.15 Å^−1^) or NaH (3.32 Å^−1^)^34^,^50^,^51^.

We consider that variable H cohesion during setting has two principal staggered sources: (A) hydration of the alumino-silicate GP GP1 (H_2_O+≡Si/Al–O–Si/Al≡→≡Si/Al–OH^−^+−OH–Si/Al≡) binding OH^−^; (B) PAA carboxylation at the interfaces (COOH↔COO^−^+H^+^) freeing H^+^ or H_3_O^+^. (A) is likely to be linked with gelation at CP, and (B) with subsequent chelation of Al at the ISP that follows at the interfaces. This is consistent with the first minima (binding of OH^−^) and maxima (release of H_3_O^+^) in *K*_C_^H^ with setting time (Fig. 3e). Likewise, the subsequent maximum in *K*_C_^F^ corresponds to low atomic cohesion, which can be associated with release of F^−^. The considerable quantity of F^−^ in the glass substantiates reports of AlO_3_F centres charge balanced by F–Ca and F–Na (ref. 36), which in G338 probably originate from both GP2 and GP3 GPs (Fig. 2a). Accordingly, we interpret oscillatory changes in *K*_C_^F^ with setting time principally with the transformation of oxyfluorides into oxides at the interfaces, accompanied by the release of F^−^ when the atomic cohesion is least (Fig. 3e). Indeed, the similarity of *K*_C_^F^ to *K*_C_^Al^ (Fig. 3e,f) points to F^−^ and Al^3+^ being partners in the same process, AlO_3_F converting to AlO_4_ at the ISP. The leaching of F^−^ during early setting provides caries resistance while actively stimulating tooth tissue remineralization, as is often claimed in the literature^7^,^11^,^37^. It seems likely, too, that the compositional species from the Ca, F+P-rich GP (GP3), which assists tooth mineralization^10^, will be released during the corrosion of the glass at CP. Finally, because oxygen is involved in hydration, fluorination and chelation, we expect setting variations in *K*_C_^O^ will closely follow the overall fracture toughness *K*_C_^av^, which indeed appears to be the case, including the ISZ maximum close to ∼15 h (Fig. 3c,f).

### Atomic structure and interfacial stress

Time-averaged structure factors *S*(*Q*) (where *Q* is the neutron scattering vector) and total radial distribution functions *G*(*r*) (where *r* is the interatomic distance, with nearest-neighbour (NN) and next NN (NNN)) for the GIC, glass and polymer components measured separately are compared in Fig. 4a,b. *S*(*Q*) for the GIC and polymer include artefacts in amplitude, relating to the featureless, but substantial, incoherent scattering of hydrogen (Methods). The peak positions on *Q* axis in *S*(*Q*) and in *G*(*r*), though are reliable. Deuteration was avoided in this study, because this would have adversely affected diffusion and cement-setting processes.

Both GIC and glass *S*(*Q*)s exhibit an initial peak—known as the first sharp diffraction peak (FSDP). This directly reflects the average length of intermediate-range order domain *r*_IRO_ in oxide glasses^42^. The position of the FSDP *Q*_FSDP_ (2*π*/*r*_IRO_) is composition- and pressure dependent. The fluctuating position of the FSDP (Fig. 4c,d) therefore indicates changes in composition and internal stress. *Q*_FSDP_ for G338 glass is located between 1.5 Å^−1^ for silica and alumina-silicate glasses and 1.9 Å^–1^ for phosphate glasses^42^. We therefore attribute the initial decrease in *Q*_FSDP_ for GIC (Fig. 4d) to the release of phosphate from the Ca, F+P-rich GP (GP3 Fig. 2a). Beyond the ISP there is a sharp increase consistent with compression developing in the remaining glass, followed by decompression, defining the start and end of ISZ. The internal pressure in the glass can be estimated by comparing the shift in *Q*_FSDP_ for silica with the permanent density change under pressure^52^, and points to ∼1 GPa being generated in the glass after the ISP at ∼8 h, then released by ∼24 h (Fig. 4d).

The integrated small-angle neutron scattering (SANS) from 0.5 to 1 Å^–1^ (
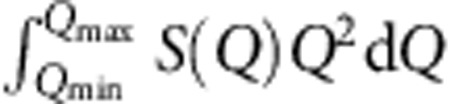
) from Fig. 4c is also shown in Fig. 4d. SANS, which measures differential density at the nano-level, is dominated here (∼0.5 Å^–1^) by Porod scattering from interfaces^42^, which in this case will be principally between the polymer and glass components (Fig. 4c). As the major amorphous phase separation structure in the glass (GP_2_) is ∼50 nm in size (Fig. 2a), equivalent to ∼0.002 Å^–1^, it is out of range for the present SANS experiments. Instead SANS intensity, which is proportional to interfacial density within the GIC, increases towards the ISZ and then drops sharply before recovering at ∼24 h; this indicates initial tension at the hybrid interfaces when the density is lowest, then stress release over the ISZ when the original density recovers (Fig. 4d).

Taken together, these variations in nanoscopic structure start with decreased density contrast pointing to tension at the interfaces (SANS) as the glass compresses (increase in FSDP *Q*_FSDP_) constrained by the increasingly rigid surrounding matrix. This is followed by overall stress release, where density contrast at the interfaces observed from SANS and pressure from the decrease in *Q*_FSDP_ is restored, and suggests interfacial failure develops over the later stages of the ISZ. Indeed, indentation studies have shown that macroscopic cracks occur at GIC hybrid interfaces^13^. Potential failure should occur via Al^3+^ linkages, as an alternative to fracture propagation at ionic sites within the polysalt complex, which has also been suggested^48^, where Al^3+^ bonding may also play an important role.

To ascertain whether complementary changes in real-space structure occur, we have turned to time-dependent total radial distribution functions, *G*(*r*)s (Fig. 4e). Because the hydrogen content of the polymer and GIC distorts amplitudes, conventional pair-distribution analysis was replaced by differencing out the effects of hydrogen in the setting of GIC by using Δ*G*(*r*)s (Fig. 4f). Furthermore, Δ*G*(*r*)s also masks the unreacted glass and polymer components, highlighting changes in local atomic structure at the interfacial regions during cementation.

Separately measured *G*(*r*)s for GIC, glass and polymer exhibit two well-resolved peaks, due to NN and NNN interatomic distances, respectively (Fig. 4b). This complex multicomponent composite includes numerous interatomic distances within the NN and NNN envelopes, related both to the polymer and glass, as well as to the organic–inorganic interfaces. Figure 4e is therefore annotated with the principal atomic pair correlations in the polymer, such as C=O and C–C, and Al–O_4_, Al–O_6_ and O–[Si/Al]–O in the G338 glass; as well as fluorinated environments identified spectroscopically^36^, including F–Ca and F–Na. None of these can be unequivocally identified.

Measurable changes occur in *G*(*r*) with setting, however, enhanced with differencing, which effectively removes unreacted components in the bulk (Fig. 4e,f). Notably, the coordination of interfacial Al(IV)O transforms to Al(VI)O over the ISZ, with complementary fluctuations in the carboxylate (C=O) correlations, coinciding with generation and then release of internal interfacial stress (Fig. 4d,f). In addition, the switch between Ca/Na–F and Ca/Na–O is consistent with discharge of F (Fig. 3e). Both swings in nanoscopic structure–SANS and FSDP (Fig. 4d) and in atomic structure—Δ*G*(*r*) (Fig. 4f), coincide with the rise and fall of toughness *K*_C_^av^ around 15 h (Fig. 3c) and the complementary minimum in atomic cohesion (Fig. 3a).

## Discussion

Comparatively few materials have been measured to date using NCS—GICs being the first complex and dynamic system to be studied. Necessarily, there are differences in NCS profiles with temperature, pressure and interatomic bonding, the latter having provided the avenue to track changing atomic cohesion and dynamic interfacial configurations during cementation. Comparisons between elemental NCS peak widths Δ*p*_i_ (Supplementary Fig. 3a) and overall Δ*p* (Supplementary Fig. 3b) and fracture toughness therefore need to relate to the specific materials studied—glass, GIC or polymer—and for them to be measured under the same conditions.

Such geometric switching, mainly dynamic configurations of Al ranging from four-coordinate tetrahedra originating in the glass to a five-coordinate pyramidal, is the likely initial GIC-setting sequence between CP and the ISP. The coherent THz frequencies detected at the developing hybrid interface are in the collective mode range, comprised of twisting, rocking, flexing and compressing modes—potentially at flexural Al pivots. Importantly, these fall among the collective modes reported for SiO_2_ (ref. 44), inorganic glasses and zeolite structures^44^, and typical of the dynamics of two-level systems^42^.

The successful juxtaposition of *in situ* sub-THz spectroscopy and neutron methods with DSC herein has enabled the complex setting-reaction processes of GIC dental composites to be unravelled structurally, energetically and dynamically at the atomic level for the first time. Our methods are equally applicable to studying fracture-toughness development in modified bioactive cement compositions^53^, where setting may be faster or slower, or in resin-modified GICs before and after curing, including the collective THz dynamics, which, in turn, are linked to inter-particle and interfacial dynamics. We expect that this battery of techniques will also offer advantages more generally for studying mechanical toughness microscopically and non-destructively in other types of mercury-free cements during setting.

## Methods

### Materials

The composition of the G338 glass powder (First Scientific Dental GmbH, Elmsohm, Germany) was Na_6.3_Ca_6.6_P_6.2_Al_16.9_Si_11.8_O_32.5_F_19.7_ (ref. 7) and was hand-mixed with Chemflex liquid (Dentsply DeTrey GmbH, Germany) of 40% polyacrylic acid solution, in a respective 2.5:1 ratio for all experiments (Fig. 1b), the proportions leaving the aqueous polymer fraction above the percolation threshold (16%), ensuring that glass particles were fully enveloped. Fresh cement was promptly loaded into measurement canisters. Setting time was recorded from the start of mixing. In each case, the powder and polymer were similarly measured separately, the 2.5:1 ratio providing the baseline *t*=0 values (Fig. 3a,c).

### Transmission electron microscopy

TEM was performed at the NanoVision Centre at QMUL using scanning electron microscopy and TEM facilities. The G338 glass powder was dried before mounting. With an average particle size range of ∼4 μm (Supplementary Fig. 4), many smaller fragments of ∼1 μm were also imaged, revealing phase separation of 50-nm rosette globules within a matrix (Fig. 2a).

### Differential scanning calorimetry

The isobaric heat capacity (*C*_p_) data for both G338 and the GIC sample subjected to the 62 h setting and subsequent first DSC up- and downscans (Fig. 2b) were collected in an argon atmosphere using a Netzsche STA449C; reproducibility being checked for baseline drift. G338 underwent two runs of up- and downscan (see glass 338 upscan-1 and upscan-2 curves). For scan 1, the sample was held for 5 min at 323 K, heated at 20 K min^−1^ to 873 K and then cooled back to 523 K at 20 K min^−1^. Scan 2 followed the same procedure. *C*_p_ was determined using a sapphire reference.

Supplementary Fig. 1 shows two upscan curves for both the DSC output (isobaric heat capacity *C*_p_) and the mass change of the GIC sample, which was subjected to a 62-h setting. The sample was upscanned in argon at 20 K min^−1^. The first upscan curve in Supplementary Fig. 1a displays two endothermic responses indicating the water evaporation between 340 and 600 K, and the PAA decomposition between 600 and 800 K. These two thermal responses are reflected in the two-stage drop in mass as shown in Supplementary Fig. 1b.

### Coherent terahertz spectroscopy

Spectra of G338, Chemflex and the setting cement (Fig. 2c) were obtained using a unique coherent THz transceiver^33^ incorporating vector-network-analyser-driven quasi-optical circuitry (Supplementary Fig. 5). The cement was loaded into a THz-transparent (polyethylene) vessel (Supplementary Fig. 6) and set at the focal point of fast mirrors, F2 and sealed to maintain water content. The sample absorbance was recorded as spectral reflectance response relative to a standard flat aluminium reflector.

### Neutron Compton scattering

NCS spectra were obtained using VESUVIO spectrometer at the ISIS neutron source, with sample sizes of 55 g glass, 20 g polymer and 14 g cements (10 g glass+4 g polymer—to give 2.5:1 mix ratios), and repeated at differing temperatures (280, 300 and 320 K). Under the conditions of high neutron energy transfer, 1–30 eV, and wavevector transfer, 30–200 Å^−1^, the impulse approximation is valid^34^. This treats the neutron scattering event as involving a single atom, with conservation of the total kinetic energy and momentum of the neutron plus the atom. NCS therefore probes the momentum distribution *n*_i_(*p*) of each element *i* present in the sample.

Calibration was first done by measuring the empty beam and the empty sample holder. The empty sample holder spectrum and the multiple scattering were always subtracted before any data analyses. Multiple scattering was calculated using the assessed Monte Carlo code DINSMS^54^, which is routinely used for multiple-scattering determinations for NCS experiments. In particular, the code calculates the time-of-flight spectrum of multiply scattered neutrons from samples of known geometry and compositions in DINS experiments. Such a contribution can be isolated in the simulation and subtracted from the experimental data; a detailed description can be found in ref. 54. The raw time-of-flight data for each single-detector spectrum were fitted using the known stoichiometry of the sample. The fitting was carried out after correction for multiple scattering and subtraction of the sample canister signal. Examples are shown in Supplementary Fig. 2 for forward and backward geometries. These include fitting to NCS momentum widths Δ*p*_i_ for GIC elements present as shown, taking advantage of the instrument's ability to probe single-particle momentum distribution^55^,^56^. The individual element-specific time-of-flight data were then transferred to momentum space, and then the peak width of each element Δ*p*_i_ was calculated in Å^–1^ as full width at half maximum (Supplementary Fig. 3), following existing procedures^34^. Also included is the difference between adjacent time slices close to the ISZ (Fig. 3c). The setting-dependent elemental momentum widths Δ*p*^H^, Δ*p*^F^, Δ*p*^O^ and Δ*p*^Al^ used to create the mechanical fracture toughness plots in Fig. 4e,f are shown in Supplementary Fig. 3a and exhibit significant variations with setting time. The overall average momentum widths (Σ_*i*_c_*i*_Δ*p*_*i*_ where *c*_*i*_ is the elemental atomic fraction) for setting at 280, 300 and 320 K, Δ*p*^280 K^, Δ*p*^300 K^, Δ*p*^320 K^, from which *K*_C_^av^ plots in Fig. 3d were obtained, are plotted in Supplementary Fig. 3b.

### Neutron diffraction

Data were collected using NIMROD at ISIS^35^. Calibration was done using a vanadium reference, after which glass powder and liquid were measured in a TiZr cell. Setting GIC measurements were performed over 24 h. Data were analysed using the Gudrun program package^57^ to correct for the contributions from the empty cell, instrument background and to normalize the data to absolute units using the scattering of a vanadium standard absorption before attenuation and multiple-scattering corrections. Correction for the contribution from inelastic scattering by the sample was made using a well-established method^58^, incorporating equations developed for total-scattering correlation functions to provide consistent definitions^59^.

The total-scattering structure factor *F*(*Q*) measured in the absolute units of barn per steradian per atom is defined as follows:


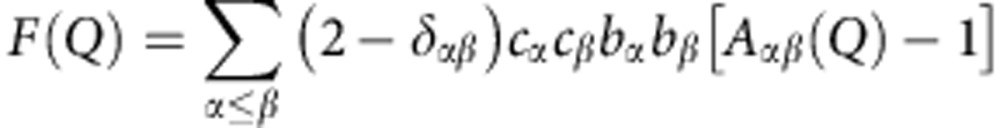


Where *c*_α_ and *c*_β_ are the concentrations of atom type α and β; *b*_α_ and *b*_β_ are their corresponding neutron scattering lengths; *δ*_αβ_ is the Kronecker delta function to avoid double-counting interactions between like-atom pairs; *Aα*_β_(*Q*) are the Faber–Ziman partial structure factors.

Structure factor *S*(*Q*) was obtained by normalizing *F*(*Q*):


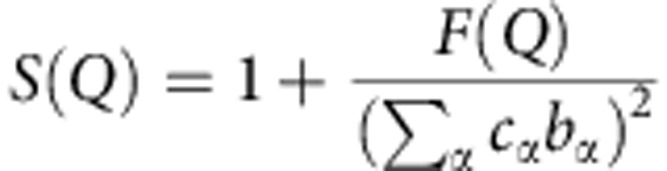


Where 
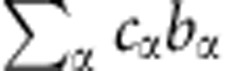
 is material scattering length.

There is a negative swing at the low-*Q* region in polymer *S*(*Q*) in Fig. 4a. This is because the scattering length of the polymer is very small, due to the negative incoherent scattering length of H, which then amplifies the negative peak in the low-*Q* region of *F*(*Q*) during normalization. This negative swing is thus not shown in Fig. 4a. This anomaly can be avoided if deuterated specimens are used, but was avoided in this study because of its affect on the dynamics of the setting process.

The total radial distribution function *G*(*r*) is obtained by the direct Fourier transform of *F*(*Q*):


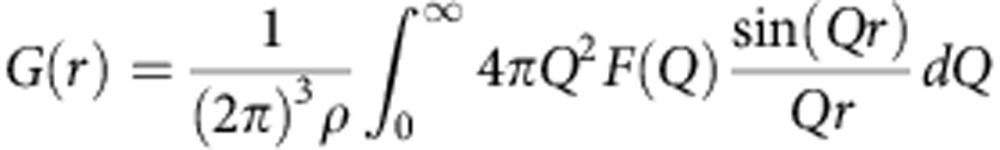


where *ρ* is the atomic density of the material.

As the samples are sealed during measurements, trends in *S*(*Q*) and *G*(*r*) for the polymer are consistent with a fixed hydrogen content. Due to the effect of high hydrogen content of the GIC, conventional pair-distribution analysis of *G*(*r*) was replaced by differencing (Fig. 4f).

## Additional information

**How to cite this article:** Tian, K. V. *et al*. Atomic and vibrational origins of mechanical toughness in bioactive cement during setting. *Nat. Commun*. 6:8631 doi: 10.1038/ncomms9631 (2015).

## Supplementary Material

Supplementary Information Supplementary Figures 1-6
