# Supplementary material for: Atomic and vibrational origins of mechanical toughness in bioactive cement during setting
Source: Nat Commun. 2015 Nov 9;6:8631. doi: 10.1038/ncomms9631 (PMC4659834; doi:10.1038/ncomms9631)
Supplement: Supplementary Information — Supplementary Figures 1-6 [file ncomms9631-s1.pdf]

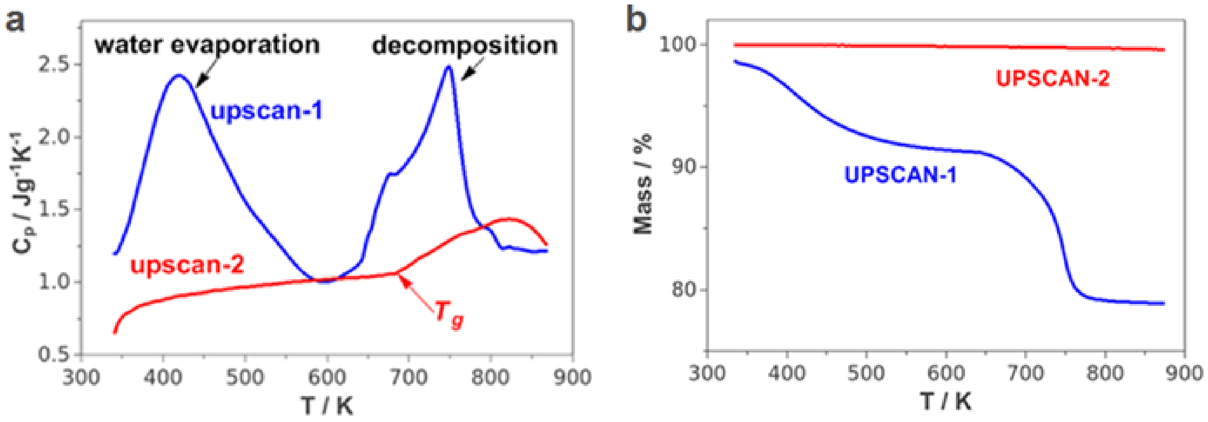

Supplementary Figure 1 | a: Isobaric heat capacity (C_p) (**a**) and mass change (**b**) as functions of temperature for the GIC sample after 62 hours setting, showing release of water and organic components.

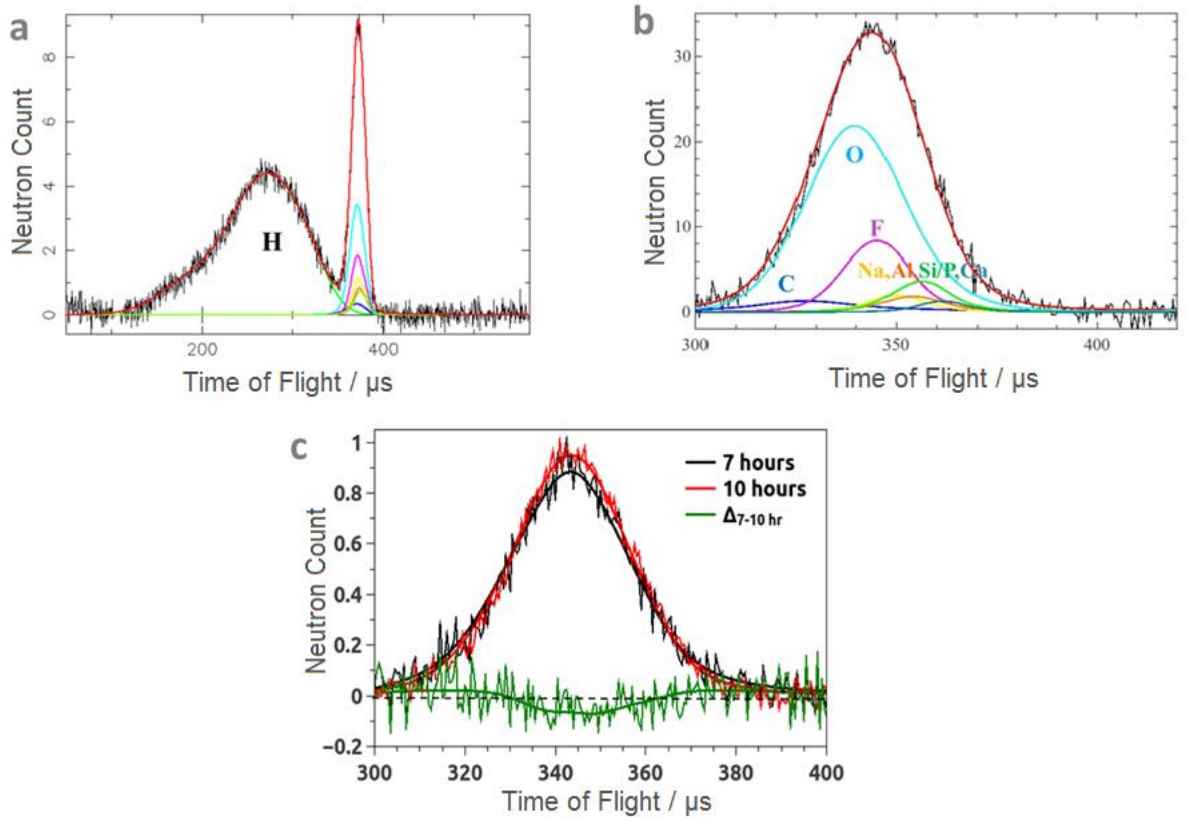

Supplementary Figure 2 | a, Example of forward scattering ToF data deconvoluted into elemental contributions by mass, each weighted by the stoichiometry. **b**, Corresponding backscattering peaks. **c**, NCS profiles (raw and fit data) of the total cement envelope at 7 and 10 hours, after initiation of setting; difference spectrum (raw and fitted data) is shown in green.

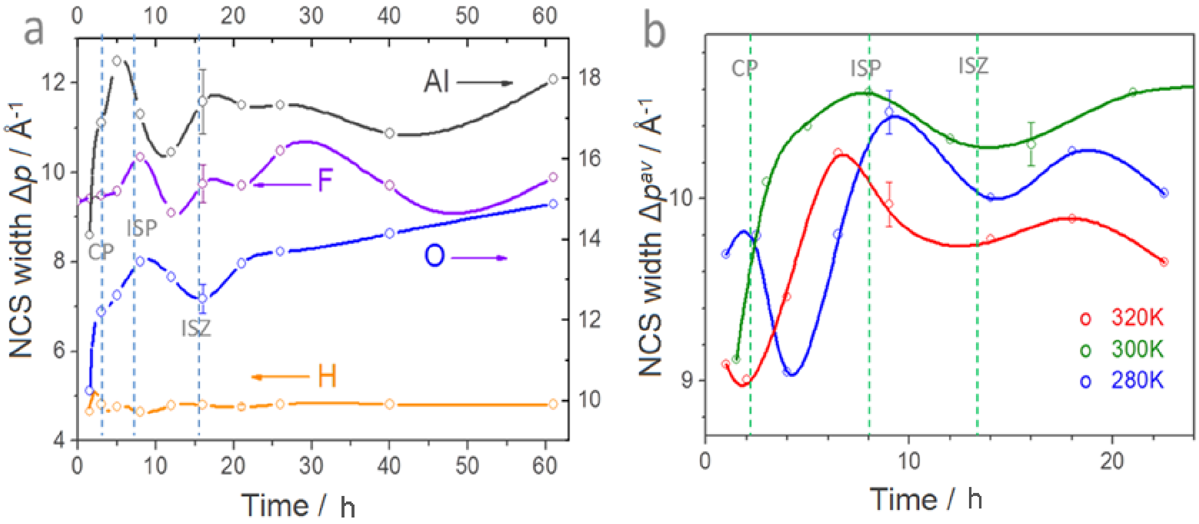

Supplementary Figure 3 | **a**, Elemental NCS peak widths Δp_i for H, O, F and Al. **b**, Average NCS peak widths $\Sigma_i c_i \Delta p_i$ for setting at 280 K, 300 K and 320 K, where c_i is the elemental atomic fraction. CP-coupling point, ISP-initial setting point, ISZ-intermediate stress zone; corresponding to early composite setting stages from Fig. 3.

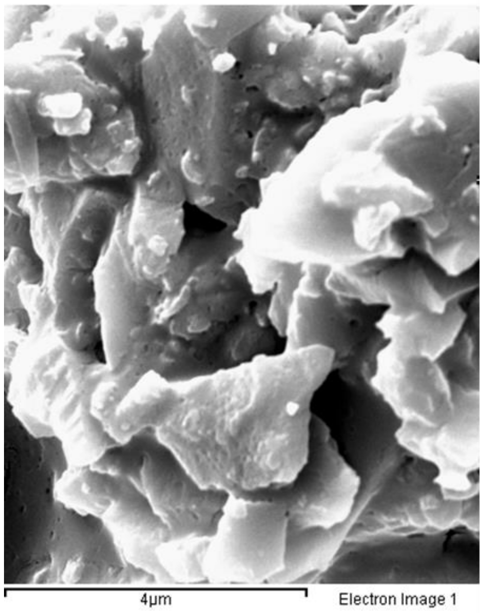

Supplementary Figure 4 | SEM image of cement glass particles showing fractured surfaces.

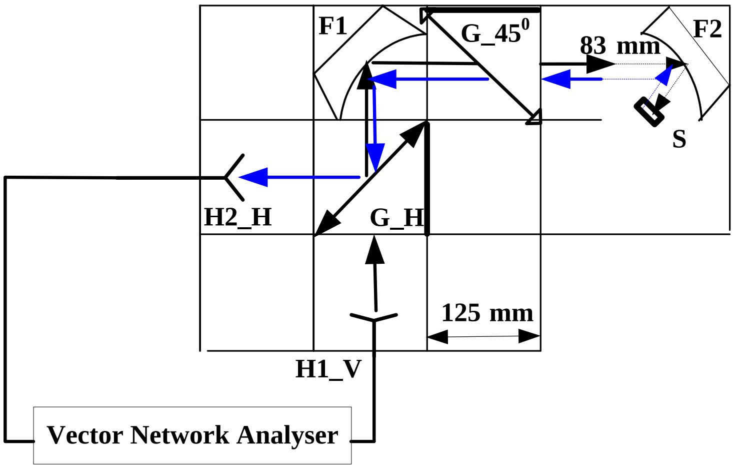

Supplementary Figure 5 | Schematic diagram of the quasi-optical transmissometer. H1 and H2 denote a pair of corrugated feed-horns transceiver and the latter capital letter V and H present the linearly-vertical and horizontal polarised electric fields, respectively; F1, is an ellipsoidal reflector with focal length 250 mm and F2, a spherical mirror with focal length 83 mm; G_H presents a wire-grid with horizontal polarization and G_45^0 stands for a wire-grid with 45^0 polarization and S is the sample under test.

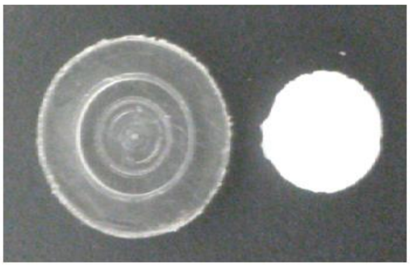

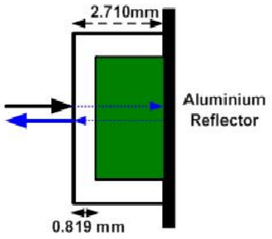

Supplementary Figure 6 | (left) Real TPX® cap and GIC; (right) the schematic plot of TPX® cap and the samples under the test are fully filled in the green area.
